# Dynamic Transitions in Neuronal Network Firing Sustained by Abnormal Astrocyte Feedback

**DOI:** 10.1155/2020/8864246

**Published:** 2020-11-22

**Authors:** Yangyang Yu, Zhixuan Yuan, Yongchen Fan, Jiajia Li, Ying Wu

**Affiliations:** ^1^State Key Laboratory for Strength and Vibration of Mechanical Structures, School of Aerospace Engineering, Xi'an Jiaotong University, Xi'an 710049, China; ^2^School of Information & Control Engineering, Xi'an University of Architecture & Technology, 710055, China

## Abstract

Astrocytes play a crucial role in neuronal firing activity. Their abnormal state may lead to the pathological transition of neuronal firing patterns and even induce seizures. However, there is still little evidence explaining how the astrocyte network modulates seizures caused by structural abnormalities, such as gliosis. To explore the role of gliosis of the astrocyte network in epileptic seizures, we first established a direct astrocyte feedback neuronal network model on the basis of the hippocampal CA3 neuron-astrocyte model to simulate the condition of gliosis when astrocyte processes swell and the feedback to neurons increases in an abnormal state. We analyzed the firing pattern transitions of the neuronal network when astrocyte feedback starts to change via increases in both astrocyte feedback intensity and the connection probability of astrocytes to neurons in the network. The results show that as the connection probability and astrocyte feedback intensity increase, neuronal firing transforms from a nonepileptic synchronous firing state to an asynchronous firing state, and when astrocyte feedback starts to become abnormal, seizure-like firing becomes more severe and synchronized; meanwhile, the synchronization area continues to expand and eventually transforms into long-term seizure-like synchronous firing. Therefore, our results prove that astrocyte feedback can regulate the firing of the neuronal network, and when the astrocyte network develops gliosis, there will be an increase in the induction rate of epileptic seizures.

## 1. Introduction

Epilepsy is one of the most common neurological diseases, affecting nearly 70 million people worldwide, and it is characterized by the aberrant synchronous firing of neurons [1–3]. It is generally believed that the reason for the aberrant synchronous firing of neurons is the imbalance between synaptic excitability and inhibition [4–6], which is caused by changes in the structure and function of neurons themselves, including changes in neurotransmitters and mutations in receptors, ion channels, and ion transporters, and alterations in network topology [7]. However, decades of studies have found that the abnormal feedback effect of astrocytes on neurons has a critical effect on the balance of neuronal excitability and inhibition and can even cause epilepsy [8–10].

Astrocytes are the most important type of glial cells in the central nervous system (CNS) and play a crucial role in maintaining the functional stability of the CNS [11, 12]. Functionally, astrocytes can regulate chemicals in the extracellular space [13] and maintain the steady state of ion concentrations in the extracellular space [14–17]. And astrocytes can respond to the stimulation of neuronal activity and release glial transmitters to regulate neuronal firing, and the concept of “tripartite synapse” is proposed to describe the bidirectional communication between astrocytes and neurons [18]. At present, many physiological experiments have shown that when astrocytes have abnormal functioning, this will lead to seizure-like events such as the aberrant synchronous firing of neurons [10, 17, 19, 20], and through mathematical modeling methods, many researchers have studied the possibility of dysfunctional astrocytes participating in neuronal epilepsy [21–28]. For instance, Amiri et al. studied the effect of Ca^2+^ oscillations in astrocyte clusters on the firing of neuronal populations by constructing a “tripartite synapse” network model in the hippocampus, suggesting that astrocytes can affect the firing activity of neurons [29, 30]. Fan et al. proposed a computational mathematical model that can be used to study synchronized seizure behavior [21]. Du et al. studied the effect of the K^+^ concentration on neuronal firing in epilepsy and the different energy requirements of neurons in normal and epileptic states by constructing a coupling model of astrocytes and neurons in the hippocampus [26]. Li and Rinzel proposed an improved coupling model of astrocytes and neurons, revealing that seizure-like firing occurs when astrocytes degrade glutamate abnormally [27]. However, few studies have investigated the effect of astrocyte structural abnormalities on neuronal epilepsy by modeling methods.

In fact, the structure of astrocytes plays an important role in the CNS and can provide structural support and an energy supply for neurons [31, 32]. In recent years, physiological experiments have shown that an abnormal astrocyte structure can also cause seizure-like events [33, 34]. One typical example of this is gliosis, which is an important factor causing abnormal astrocyte structure, leading to morphological and physiological variations in astrocytes after CNS injury [35]. Gliosis can cause abnormal feedback from astrocytes to neurons and induce epilepsy. Indeed, gliosis has been found to be a hallmark of epilepsy [7, 33, 34]. Therefore, we introduced a new model based on the actual coupling structure of neuron-astrocytes to investigate the effect of gliosis on epilepsy.

In the actual brain, a single astrocyte can be connected to numerous neurons and up to 2,000,000 synapses [36, 37], and a neuron can be surrounded by different astrocytes [38], thereby forming a complex coupling network [39–41]. Based on Amiri's model, we proposed a feedback model from astrocytes to neurons, which is constructed according to the actual coupling structure of astrocytes and neurons on the basis of the hippocampal CA3 [38–42]. Compared with Amiri's functional model and most of the coupling model of neuron-astrocytes, our model more accurately emulates the physiological structure in terms of topology and spatial distribution and can simulate the structural changes of the astrocyte population, which has profound physiological significance. We used 50 pyramidal neurons and 50 interneurons to establish a neuronal network with synaptic connections. An astrocyte network was formed by 50 astrocytes connected by gap junctions; finally, based on the “tripartite synapse” model, neurons and astrocytes were connected to form a coupling network.

In this work, we mainly studied the possible mechanism of epilepsy under simulated gliosis conditions by developing a mass network model between neurons and astrocytes with an updated feedback model from astrocytes to neurons. The effect of excitatory conductance, the connection probability between astrocytes and neurons, and the astrocyte feedback intensity on the synchronization of pyramidal neuron populations were considered separately, and the firing activity of the pyramidal neuronal population was analyzed. Then, we introduced the concept of energy consumption to characterize the transition of neuronal firing patterns. The study was further expanded by adding electrical synapses to the model to verify the robust effect of neuronal astrocyte feedback on neuronal firing. Finally, by analyzing the numerical simulation results, the effect of the feedback mechanism of astrocytes on the transition of the firing pattern of the neuronal network was summarized, and the possible mechanism of seizures was discussed.

## 2. Model and Methods

### 2.1. Coupling Network Model of Neurons and Astrocytes

In this paper, we constructed a neural network consisting of pyramidal neurons and interneurons, and each neuron is described by the modified Morris-Lecar model [43]. The membrane potentials of *v*_*i*_^*PV*^ and *v*_*i*_^IN^ for pyramidal neurons and interneurons are as follows [30]:

(1) $$\begin{array}{c} C_{m}{\dot{v}}_{i}^{PV}\left(t\right)=I_{i}^{PY}\left(t\right)-\left(g_{Ca}m_{\infty }\left(v_{i}^{PY}\left(t\right)\right)\left(v_{i}^{PY}\left(t\right)-v_{Ca}\right)+g_{K}w_{i}^{PY}\left(\text{t}\right)\left(v_{i}^{PY}\left(t\right)-v_{K}\right)+g_{L}\left(v_{i}^{PY}\left(t\right)-v_{L}\right)\right),\\ {\dot{w}}_{i}^{PV}\left(t\right)=\emptyset \frac{w_{\infty }\left(v_{i}^{PV}\left(t\right)\right)-w_{i}^{PY}\left(\text{t}\right)}{\tau_{w}\left(v_{i}^{PV}\left(t\right)\right)},\\ C_{m}{\dot{v}}_{i}^{\text{IN}}\left(t\right)=I_{i}^{\text{IN}}\left(t\right)-\left(g_{Ca}m_{\infty }\left(v_{i}^{\text{IN}}\left(\text{t}\right)\right)\left(v_{i}^{\text{IN}}\left(t\right)-v_{Ca}\right)+g_{K}w_{i}^{\text{IN}}\left(t\right)\left(v_{i}^{\text{IN}}\left(t\right)-v_{K}\right)+g_{L}\left(v_{i}^{\text{IN}}\left(t\right)-v_{L}\right)\right)\\ {\dot{w}}_{i}^{\text{IN}}\left(t\right)=\emptyset \frac{w_{\infty }\left(v_{i}^{\text{IN}}\left(t\right)\right)-w_{i}^{\text{IN}}\left(\text{t}\right)}{\tau_{w}\left(v_{i}^{\text{IN}}\left(t\right)\right)} \end{array}$$

where *v*_*i*_^*PV*^ and *v*_*i*_^IN^ represent the membrane potential of the *i*th pyramidal neuron and the *i*th interneuron, respectively; ${\dot{w}}_{i}^{PV}$ and ${\dot{w}}_{i}^{\text{IN}}$ are the restoration variables and represent the ratio of the number of open potassium channels to the number of excitatory pyramidal neurons;*g*_*Ca*_, *g*_*K*_, and *g*_*L*_ represent the channel conductance of the Ca^2+^, K^+^, and leak current, respectively, which play an important role in forming the membrane potential; *v*_*Ca*_ and *v*_*K*_ are the Nernst potentials of Ca^2+^ and K^+^, respectively; *v*_*L*_ is the reversal potential of the neuronal leak channels; ∅ is the temperature parameter, which is constant; and*m*_∞_(*v*), *w*_∞_(*v*), and *τ*_*w*_(*v*) (*v* = *v*_*i*_^*PV*^(*t*)or*v*_*i*_^IN^(*t*)) describe the role of voltage-dependent ion channels in the membrane potential and are given by

(2) $$\begin{array}{c} m_{\infty }\left(v\right)=\frac{1}{2}\left[1+\tan h\left(\frac{v-v_{1}}{v_{2}}\right)\right],\\ w_{\infty }\left(v\right)=\frac{1}{2}\left[1+\tan h\left(\frac{v-v_{3}}{v_{4}}\right)\right],\\ \tau_{w}\left(v\right)=\frac{1}{\cos h\left(v-v_{1}/2v_{4}\right)}. \end{array}$$

*I*_*i*_^*PY*^(*t*) and *I*_*i*_^IN^(*t*) act on pyramidal neurons and interneurons, respectively, which are affected by external constant input currents *I*_const,*i*_^*x*^(*t*), system noise currents *I*_noise,*i*_^*x*^(*t*), slowly varying currents *I*_slow,*i*_^*x*^(*t*), synaptic currents from adjacent pyramidal neurons and interneurons *I*_syn,*i*_^*x*^(*t*), and feedback currents from adjacent astrocytes *I*_as,*i*_^*x*^(*t*) (*x* = *PY*, IN). The specific forms of *I*_*i*_^*PY*^(*t*) and *I*_*i*_^IN^(*t*) are as follows:

(3) $$\begin{array}{c} I_{i}^{PY}\left(t\right)=I_{\text{const},i}^{PY}\left(t\right)+I_{\text{noise},i}^{PY}\left(t\right)+I_{\text{slow},i}^{PY}\left(t\right)+I_{\text{syn},i}^{PY}\left(t\right)+I_{\text{as},i}^{PY}\left(t\right),\\ I_{i}^{\text{IN}}\left(t\right)=I_{\text{const},i}^{\text{IN}}\left(t\right)+I_{\text{noise},i}^{\text{IN}}\left(t\right)+I_{\text{slow},i}^{\text{IN}}\left(t\right)+I_{\text{syn},i}^{\text{IN}}\left(t\right)+I_{\text{as},i}^{\text{IN}}\left(t\right),\\ {\dot{I}}_{\text{slow},i}^{x}\left(t\right)=\varepsilon \left(v^{*}-v_{i}^{x}\left(t\right)-\alpha I_{\text{slow},i}^{x}\left(t\right)\right),\left(x=\text{PY},\text{IN}\right). \end{array}$$

where *ε* and *α* are the variables that control the bursting behavior of neurons, and *v*^∗^ is a factor driving the generation of bursting.

In our model, neurons are connected to each other through chemical synapses, wherein pyramidal neurons are excitatory neurons with unidirectionally connected excitatory synapses. Interneurons are inhibitory neurons, and they are connected by inhibitory synapses. Pyramidal neurons stimulate the activity of interneurons, and the interneurons inhibit the activity of pyramidal neurons, which combine to form a bidirectional connection. This whole process is mainly achieved through neurotransmitter transmission, which is dependent on the membrane potential of each neuron. The concentration of neurotransmitter in the synaptic cleft that is released from the *i*th neuron (presynaptic neuron) is described as follows:

(4) $$\begin{array}{c} {\left[\text{T}\right]}_{i-1}^{x}=\frac{1}{1+\exp\left(-\left(v_{i-1}^{x}-\theta_{s}\right)/\sigma_{s}\right)},\left(x=\text{PY},\text{IN}\right), \end{array}$$

where *θ*_*s*_ is the half-activation voltage and *σ*_*s*_ is the steepness of the sigmoid function. The synaptic currents are modulated by synaptic variables g_*i*_^*x*^(*x* = PY, IN), and the equation is as follows [44]:

(5) $$\begin{array}{c} {\dot{g}}_{i}^{x}\left(\text{t}\right)=\alpha_{s}{\left[\text{T}\right]}_{i-1}^{x}\left(1-g_{i}^{x}\left(t\right)\right)-\beta_{s}g_{i}^{x}\left(t\right),\left(x=PY,\text{IN}\right), \end{array}$$

where ${\dot{g}}_{i}^{x}\left(\text{t}\right)$ refers to the open level of neuroreceptors and *α*_*s*_ and *β*_*s*_ are the rate constants that determine the increase and decrease in ${\dot{g}}_{i}^{x}\left(\text{t}\right)$, respectively. Consequently, the specific form of the synaptic currents is as follows [30]:

(6) $$\begin{array}{c} I_{\text{syn},i}^{PY}\left(t\right)=g_{\text{se}}g_{i-1}^{PY}\left(\text{t}\right)\left(v_{i}^{PY}\left(t\right)-v_{\text{se}}\right)+g_{si}\left(g_{i-1}^{\text{IN}}\left(\text{t}\right)+g_{i}^{\text{IN}}\left(\text{t}\right)\right)\left(v_{i}^{PY}\left(t\right)-v_{si}\right),\\ I_{\text{syn},i}^{\text{IN}}\left(t\right)=g_{\text{se}}g_{i}^{PY}\left(\text{t}\right)\left(v_{i}^{\text{IN}}\left(t\right)-v_{\text{se}}\right)+g_{\text{si}}g_{i-1}^{\text{IN}}\left(\text{t}\right)\left(v_{i}^{\text{IN}}\left(t\right)-v_{\text{si}}\right), \end{array}$$

where *g*_se_ and *g*_si_ are the conductance of the excitatory synapses and inhibitory synapses, respectively, and *v*_se_ and *v*_si_ are the excitatory and inhibitory equilibrium potentials, respectively.

After neurotransmitters are released into the synaptic cleft, some neurotransmitters bind to receptors on adjacent astrocytes, causing Ca^2+^ oscillations in astrocytes. To describe the dynamics of this process, we used the improved Li-Rinzel model [30, 45, 46]. The mathematical forms are as follows:

(7) $$\begin{array}{c} \left[\overset{˙}{\text{C}{\text{a}}^{2+}}\right]=J_{\text{chan}}+J_{\text{leak}}+J_{\text{pump}}, \end{array}$$

(8) $$\begin{array}{c} \left[\text{I}{\dot{\text{P}}}_{3}\right]=\frac{\left({\text{IP}}_{3}^{*}-\left[{\text{IP}}_{3}\right]\right)}{\tau_{{ip}_{3}}}+r_{{ip}_{3}}\left({\left[\text{T}\right]}^{PY}+{\left[\text{T}\right]}^{\text{IN}}\right), \end{array}$$

(9) $$\begin{array}{c} \dot{q}=\alpha_{q}\left(1-q\right)-\beta_{q}q, \end{array}$$

where

(10) $$\begin{array}{c} J_{\text{chan}}=-c_{1}V_{1}p_{\infty }^{3}n_{\infty }^{3}q^{3}\left({\left[{\text{Ca}}^{2+}\right]}_{\text{ER}}-\left[{\text{Ca}}^{2+}\right]\right),\\ J_{\text{leak}}=-c_{1}V_{2}\left({\left[{\text{Ca}}^{2+}\right]}_{\text{ER}}-\left[{\text{Ca}}^{2+}\right]\right),\\ J_{\text{pump}}=-\frac{V_{3}{\left[{\text{Ca}}^{2+}\right]}^{2}}{{\left[{\text{Ca}}^{2+}\right]}^{2}+k_{3}^{2}}, \end{array}$$

where

(11) $$\begin{array}{c} p_{\infty }=\frac{\left[{\text{IP}}_{3}\right]}{\left[{\text{IP}}_{3}\right]+d_{1}},\\ n_{\infty }=\frac{\left[{\text{Ca}}^{2+}\right]}{\left[{\text{Ca}}^{2+}\right]+d_{5}},\\ \alpha_{q}=a_{2}d_{2}\frac{\left[{\text{IP}}_{3}\right]+d_{1}}{\left[{\text{IP}}_{3}\right]+d_{3}},\\ \beta_{q}=a_{2}\left[{\text{Ca}}^{2+}\right],\\ {\left[{\text{Ca}}^{2+}\right]}_{\text{ER}}=\frac{c_{0}-\left[{\text{Ca}}^{2+}\right]}{c_{1}}, \end{array}$$

where [IP_3_] is the concentration of IP_3_ in astrocytes, IP_3_^∗^ is the reversal concentration of IP_3_,*τ*_*ip*_3__ is the relaxation time constant, *r*_*ip*_3__ refers to the rate of the increase in IP_3_, [Ca^2+^] is the concentration of Ca^2+^ in the cytosol of astrocytes, and *q* refers to the proportion of activated IP_3_ receptors. *J*_chan_, *J*_leak_, and *J*_pump_ represent the calcium flux from the channel, the leakage, and the pump, respectively, and V1, V2, and V3 represent the flux rate of the corresponding calcium flux. [Ca^2+^]_ER_ is the concentration of Ca^2+^ in the ER of astrocytes.

With the action of neurotransmitters, Ca^2+^ in astrocytes oscillates and causes the release of gliotransmitters into synapses to regulate neuronal activity [15, 47]. According to the work of Volman and colleagues [46], we used a kinetic variable *f* to describe the astrocyte-neuron interaction, which has the following form:

(12) $$\begin{array}{c} \dot{f}=\frac{-f}{\tau_{{\text{Ca}}^{2+}}}+\left(1-f\right)\kappa \Phi \left(\left[{\text{Ca}}^{2+}\right]-{\left[\text{Ca}\right]}_{th}\right). \end{array}$$

In recent years, numerous morphological studies have shown that astrocytes form a network via gap junctions [48, 49]. Studies have shown that IP_3_ is the main messenger that diffuses throughout the astrocyte network through gap junctions [29, 50], so we used a simplified model to describe gap junctions:

(13) $$\begin{array}{c} J_{G,i}=k_{g}\left({\left[{\text{IP}}_{3}\right]}_{i+1}+{\left[{\text{IP}}_{3}\right]}_{i-1}-2{\left[{\text{IP}}_{3}\right]}_{i}\right), \end{array}$$

where *k*_*g*_ is a coupling coefficient representing the coupling coefficient of gap junctions; the coupling model will ultimately be added to equation (8).

**Figure 1:**
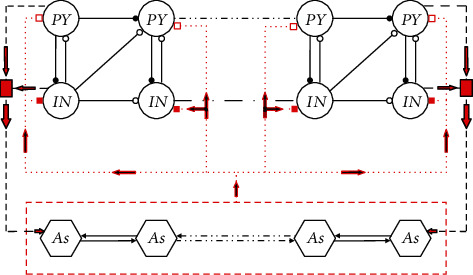
Coupling network model of neurons and astrocytes. PY, IN, and As represent the pyramidal neurons, interneurons, and astrocytes, respectively. Neurons are connected by synapses; filled circles represent excitatory synapses, empty circles represent inhibitory synapses, and red rectangles represent the collective effect of pyramidal neurons and interneurons on astrocytes. Astrocytes are connected by gap junctions; the inhibitory feedback from astrocytes to each pyramidal neuron is represented by hollow squares, and the excitatory feedback from astrocytes to each interneuron is represented by solid squares.

According to the previous description, the astrocyte network wraps around neurons to form a complex network. To describe this physiological structure, we proposed a coupling model of neurons and astrocytes. The specific form is shown in Figure 1. In this model, each neuron receives feedback from the entire astrocyte network; according to numerous physiological experimental observations, the feedback effect of astrocytes inhibits pyramidal neuron excitability [51] but enhances interneuron excitability [52]. The equation for describing the feedback effect is as follows:

(14) $$\begin{array}{c} I_{\text{as},i}^{PY}\left(t\right)=-\gamma_{1}\overset{50}{\underset{j=1}{\sum }}P_{1}f_{ij},\\ I_{\text{as},i}^{\text{IN}}\left(t\right)=\gamma_{2}\overset{50}{\underset{j=1}{\sum }}P_{2}f_{ij}, \end{array}$$

where *γ*_1_ and *γ*_2_ represent the feedback intensity from astrocytes to pyramidal neurons and interneurons, respectively. *P*_1_ and *P*_2_ are the probability of an astrocyte successfully connecting with a pyramidal neuron and an interneuron in the network, respectively, representing the degree of tightness of the connection. *f*_*ij*_ represents the interaction of the *i*th neuron and the *j*th astrocyte. To simplify the calculation, all connections in the same population have the same probability. *γ*_1_ and *P*_1_ are fixed variables.

Without special instructions, the various parameter values used in our simulation are shown in Table 1.

### 2.2. Methods

Epilepsy is characterized by synchronous seizures in neurons, and we used the synchronization of the abnormal firing of neurons as an indicator to measure seizures. To quantify the indicator, we used a cross-correlational coefficient measurement method based on the method that was used to measure the degree of synchronous firing between pairs of neurons [53]. We used *M*(*k*) and *N*(*k*) to represent the spike trains of neuronal pairs, where *k* = 1, 2, ⋯, S (*T*/*S* = *τ*; *T* is the total time interval, and *τ* is the time step). *M*(*k*) equaled 1 if the neuron fired at the *k*_*th*_ moment; otherwise, *M*(*k*) equaled 0, which was the same as *N*(*k*). The specific equation is as follows [54]:

(15) $$\begin{array}{c} \rho_{ij}=\frac{\sum_{k=1}^{S}M\left(k\right)N\left(k\right)}{\sqrt{\sum_{k=1}^{S}M\left(k\right)\sum_{i=1}^{S}N\left(k\right)}}. \end{array}$$

**Figure 2:**
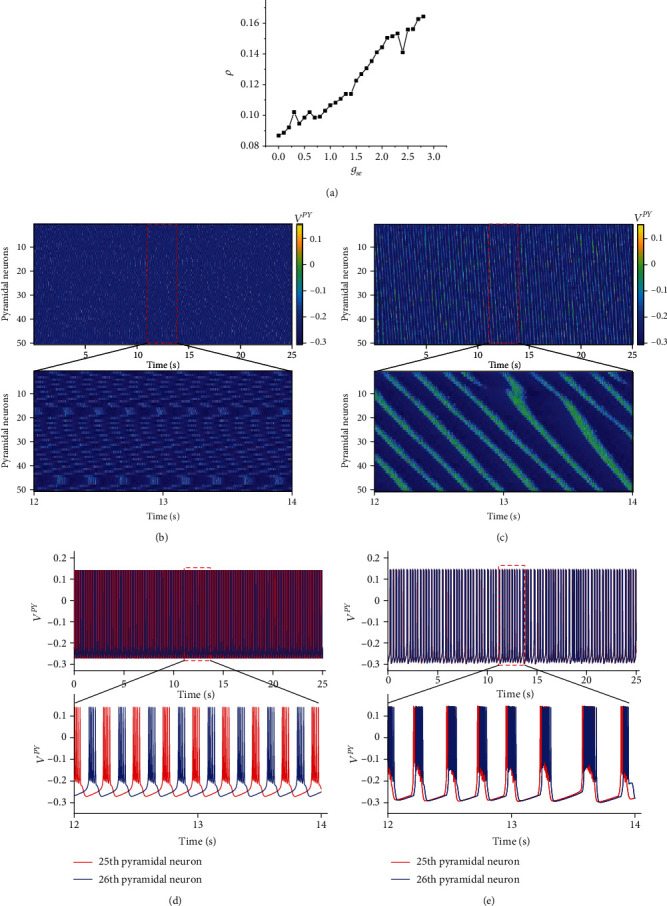
The synchronization states of the pyramidal neuronal population change with *g*_se_. (a) The correlation coefficient *ρ* of the pyramidal neuronal population changes with *g*_se_. (b) The time series of the firing of the pyramidal neuronal population at *g*_se_ = 0. (c) The time series of the firing of the pyramidal neuronal population at *g*_se_ = 2. (d) The time series of the 25th and 26th pyramidal neurons firing at *g*_se_ = 0. (e) The time series of the 25th and 26th pyramidal neurons firing at *g*_se_ = 2. The bottom of (b)–(e) shows a partially enlarged view of the corresponding figure.

The firing threshold is set to -0.1, because it can be seen from Figures 2(d) and 2(e) that -0.1 can not only calibrate the peak action potential moment of normal firing but also avoid the interference of subthreshold oscillation.

In this paper, we mainly analyzed the numerical results of pyramidal neurons. We used the sliding time window method to calculate the average correlation coefficient of all pairs of pyramidal neurons in each time window and finally averaged the correlation coefficient *ρ*_*l*_, where *l* = 1, 2, ⋯, *L* (*T*/*δ* = *L*, *δ* is the interval of the individual time window) of all time windows, to obtain the final correlation coefficient *ρ*. To solve the model equations, the Runge–Kutta method with a fixed time step of 0.01 ms was used. Considering that normal firing resumes after a period of time after the observed neuronal epileptic firing, we set the total time interval *T* of the simulation to 25 s. The interval of the individual time window *δ* was 0.25 s, and 100 time windows were used in total.

## 3. Numerical Results and Discussion

### 3.1. The Effect of *g*_se_ on Neuronal Network Synchronization

In this section, we studied the effect of the conductance of the excitatory synapses *g*_se_ on the synchronization of the pyramidal neuron population with *P*_1_ = *P*_2_ = 0. The result is shown in Figure 2.

Figure 2(a) shows that the curve of the correlation coefficient *ρ* of the pyramidal neuronal population increases with the increase in *g*_se_. We know that an increase in *g*_se_ indicates an increase in the concentration of neurotransmitters released by presynaptic neurons, which will affect the firing of postsynaptic neurons, and the connection between neurons can be strengthened. Figures 2(b) and 2(c) provide the time series of the firing of the pyramidal neuronal population for *g*_se_ = 0 and *g*_se_ = 2, respectively. The results show that the firing state of the pyramidal neuronal population changes from an asynchronous to a synchronous state with the increase in *g*_se_, and we find that there is a delay in the firing of the neuronal population, which arises from the neuronal network being connected by chemical synapses. Finally, to study the variations in the synchronization of neurons in detail, we show the firing time histories of the 25th and 26th neurons in Figures 2(d) and 2(e). The results show that the firing of the two neurons changes from asynchronous to synchronous with increasing *g*_se_.

These results suggest that the synchronization of the pyramidal neuronal population increases with increasing conductance *g*_se_.

### 3.2. The Effect of *P* on Neuronal Network Synchronization

**Figure 3:**
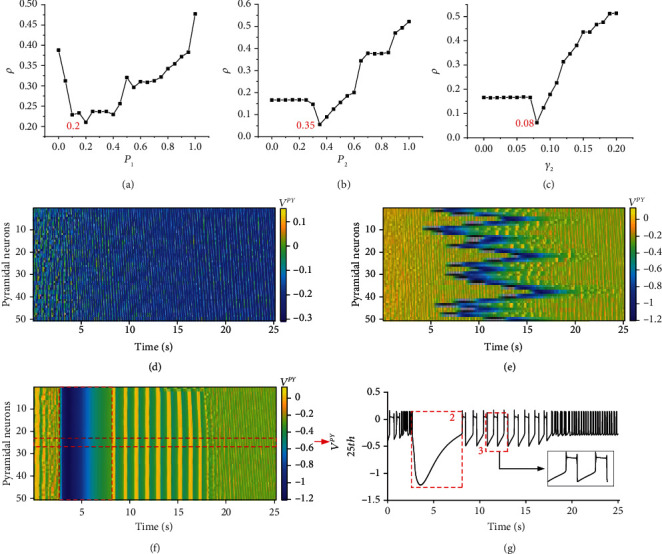
The impact of changes in *P* and *γ*_2_ on the synchronous firing of the pyramidal neuronal population. (a) The correlation coefficient *ρ* of the pyramidal neuronal population changes with *P*_1_. (b) The correlation coefficient *ρ* of the pyramidal neuronal population changes with *P*_2_. (c) The correlation coefficient *ρ* of the pyramidal neuronal population changes with *γ*_2_. (d) The time series of pyramidal neuronal population firing at *P*_2_ = 0. (e) The time series of pyramidal neuronal population firing at *P*_2_ = 0.35. (f) The time series of pyramidal neuronal population firing at *P*_2_ = 1. (g) The time series of the firing of the 25th pyramidal neuron is shown in (f).

Epilepsy is characterized by the aberrant synchronous firing of neurons. To explore the reasons for the aberrant synchronous firing of neurons, we established a new model of the coupling network of neurons and astrocytes that more closely emulates the actual physiology. Because experiments have shown that the increased excitability of interneurons is beneficial in enhancing the inhibition of the nervous system and suppressing seizures [55–57], we studied the impact of changes in the connection probability of *P*_1_ with *P*_2_ = 0.8, *P*_2_with *P*_1_ = 0.8, and the astrocyte feedback intensity *γ*_2_ from astrocytes to interneurons on pyramidal neuronal population synchronous firing with *g*_se_ = 2. Only the phase of significant seizures was selected to analyze the change in synchronization, and the phase was located approximately within the time interval from 8 s to 18 s. The results are shown in Figure 3.

First, we studied the law of the firing synchronization of the neuronal population based on the connection probability *P*_1_, *P*_2_ and the feedback intensity *γ*_2_. Figures 3(a)–3(c) show that the correlation coefficient *ρ* of the pyramidal neuronal population decreases first and then increases with the connection probability *P*_1_, *P*_2_ and feedback intensity *γ*_2_, and *ρ* is minimal when *P*_1_ = 0.2, *P*_2_ = 0.35, or *γ*_2_ = 0.08. Second, we studied in detail the influence of changes in *P*_2_ on the firing transition of pyramidal neurons. Figure 3(d) shows the abundant firing of the neuronal population. There is slight depolarization block firing in the early stage, and then due to the stability of the system, normal synchronous firing quickly resumes. Figures 3(d)–3(f) show that the firing of the neuronal population changes from a nonepileptic synchronous firing state with slight depolarization block firing (Figure 3 (d)) to asynchronous firing with slight local seizure-like firing (Figure 3(e)), and then, *ρ* continues to increase until *P*_2_ = 1. The seizure-like firing is more severe and synchronized; eventually, the firing of the pyramidal neuronal population changes from asynchronous firing (Figure 3 (e)) to seizure-like synchronous firing (Figure 3(f)). The yellow strip area in Figure 3(f) indicates that the neuronal population is in a state of seizure-like synchronous firing and then resumes normal firing later, and experiments have shown that epilepsy firing reflects the synchronous firing of the neuronal population [4, 58].

**Figure 4:**
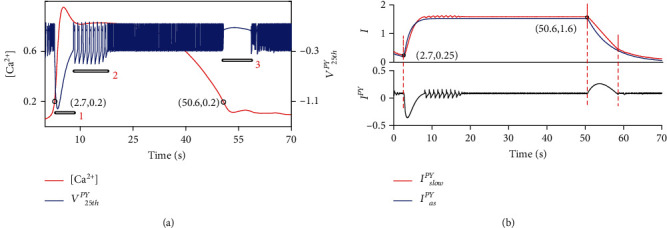
Calcium modulates neuronal epileptic firing. (a) The comparison diagram of the Ca^2+^ concentration and neuronal firing. (b) The top of (a) shows the time series of *I*_slow_^*PY*^ and *I*_as_^*PY*^, and the bottom of (a) shows the time series of *I*^*PY*^.

Figure 3(g) shows the 25th pyramidal neuron from Figure 3(f). We can observe two phenomena from Figure 3(g), which will be analyzed in Figure 4. The first phenomenon is the spreading depression in the area of box 2, which corresponds to box 1 in Figure 3(f). Studies have shown that spreading depression is closely related to epilepsy [50, 58–60]. The second phenomenon is the depolarization block in the area of box 3, which is one of the typical characteristics of epileptic seizures [3].

To investigate the structural aberrations of the astrocyte network during epileptic gliosis, which is different from the regular neighboring connection model used in previous studies [29, 30], we used an “all-to-all” connection to examine the gliosis effect on neuronal firing shown in Figure 3, where both the structural (connection probability, Figures 3(a) and 3(b)) and functional variations (feedback intensity, Figure 3(c)) reflect epileptic gliosis. The simulation results support the experimental observations that the presence of gliosis in the astrocyte network accelerates epileptic seizures [30].

Because of gliosis, reactive astrocytes become hypertrophic, and then, the processes overlap until hyperplasia produces astroglial scars [43, 61, 62]. The structural connections between astrocytes and neurons become closer and tighter, corresponding to the increase in the connection probability *P* in the model. In this process, reactive astrocytes release substances such as immunomodulators, neurotrophic factors, and growth factors to modulate the excitability of neurons. For example, cytokines (a kind of immunomodulator) released by reactive astrocytes act on neurons, which will lead to increases in postsynaptic AMPA receptors and glutamate; this causes hyperexcitability of the neuronal network and seizures [7], corresponding to the increase in the feedback intensity *γ* in the model. These results indicate that with the emergence and development of gliosis, the connection probability *P*_2_ and feedback intensity *γ*_2_ of astrocytes toward interneurons increase, resulting in an abnormal increase in feedback from astrocytes; because astrocytes are connected to each other through gap junctions, the feedback effect of the astrocytes on each neuron tends to be the same, resulting in the enhancement of the synchronization firing of the neuronal population and the continued expansion of the area of seizure-like synchronous firing. In other words, astrocytes are in gliosis, which leads to gradual increases in *P*_2_ and *γ*_2_; this causes astrocyte feedback to become abnormal, which leads to the development of seizure-like activity. The results are consistent with the observations in clinical trials that when the astrocyte network develops gliosis after severe epileptic seizures in a population of epileptic patients, seizures will be further induced and aggravated [33, 63].

Moreover, Ca^2+^ plays a vital role in the feedback from astrocytes to neurons, and studies in recent years have shown that Ca^2+^ signals are closely related to epilepsy activity [59, 64, 65]. In this work, we studied the relationship between variations in the calcium concentration [Ca^2+^] and neuronal epileptic firing by examining the 25th pyramidal neuron when the connection probability *P*_2_ = 1 (Figure 3(g)) as an example. In the models of neuronal firing, the collective currents of *I*_slow_^*PY*^ and *I*_as_^*PY*^ and the total neuronal external current *I*^*PY*^ were introduced to study the current-sensitive firing in view of neuronal firing bifurcation versus current in previous dynamical studies [54, 66]. *I*^*PY*^ is mainly regulated by *I*_slow_^*PY*^ and *I*_as_^*PY*^. To observe the regulation of Ca^2+^ completely, we extended the total time to 70 s. The result is shown in Figure 4.

Figure 4(a) shows the abundance of firing behaviors. We divided these behaviors into three main phenomena. The first phenomenon is spreading depression after high-frequency firing. The main cause of this is that after 2.7 seconds, the calcium concentration is higher than 0.2 (shown in Figure 4(a)), which causes astrocytes to release glial transmitters into nearby synapses [46] and the astrocyte feedback current *I*_as_^*PY*^ to rise rapidly and to stimulate the slow-variation current *I*_slow_^*PY*^ to also rise; however, as shown at the top of Figure 4(b), at the initial stage, the growth rate of *I*_as_^*PY*^ is greater than that of *I*_slow_^*PY*^, which then reverses. Since astrocyte feedback has an inhibitory effect on pyramidal neurons, and the slow-varying current has an excitatory effect, the competition between the two effects causes the total stimulation current *I*^*PY*^ to first become negative and then to recover (shown in the bottom of Figure 4(b)), so that the neuron appears to repolarize first and then depolarize.

Then, the self-feedback process of the slow-varying current *I*_slow_^*PY*^ leads to epileptic firing in neurons, which is process 2. In process 3, the neuron is affected by a depolarizing block. Figure 4(a) shows that the calcium concentration is lower than 0.2 after 50.6 seconds, causing astrocytes to stop releasing glial transmitters, so that *I*_as_^*PY*^ and *I*_slow_^*PY*^ decrease rapidly. The combined effect of the currents causes *I*^*PY*^ to be abnormal, leading to a depolarization block. These phenomena indicate that the transition in neuronal firing activity is regulated by Ca^2+^, which proves that abnormal astrocytes can cause neurons to exhibit epileptic firing and other abnormal behaviors.

The above results indicate that the connection probability *P* and the feedback intensity *γ* play important roles in the regulation of neuronal population synchronous firing activity and reveal the role of gliosis in the astrocyte network in the occurrence and development of epileptic seizures.

### 3.3. The Effect of *P* on Neuronal Network Energy Consumption

Neuronal epilepsy firing consumes much energy [67]. During the past few years, many researchers have studied this phenomenon [68–70] and proposed many methods to calculate the energy consumption of neurons [71–74]. To describe this feature, we used the energy consumption formula based on M-L neurons [27, 75]:

(16) $$\begin{array}{c} <H>=\frac{\left|\int_{0}^{T}H^{\prime }\left(\text{t}\right)dt\right|}{T},\\ H^{\prime }\left(\text{t}\right)=I_{i}^{PY}\left(t\right)v_{i}^{PY}\left(t\right)-\left(g_{\text{Ca}}m_{\infty }\left(v_{i}^{PY}\left(t\right)\right){\left(v_{i}^{PY}\left(t\right)-v_{\text{Ca}}\right)}^{2}+g_{K}w_{i}^{PY}\left(t\right){\left(v_{i}^{PY}\left(t\right)-v_{K}\right)}^{2}+g_{L}{\left(v_{i}^{PY}\left(t\right)-v_{L}\right)}^{2}\right) \end{array}$$

where <*H*> is the average energy consumption of the pyramidal neuronal population and *H*′(t) is the instantaneous power value of neuronal energy consumption. We mainly studied pyramidal neurons, so only the energy consumption formulas of the pyramidal neuron population are listed.

**Figure 5:**
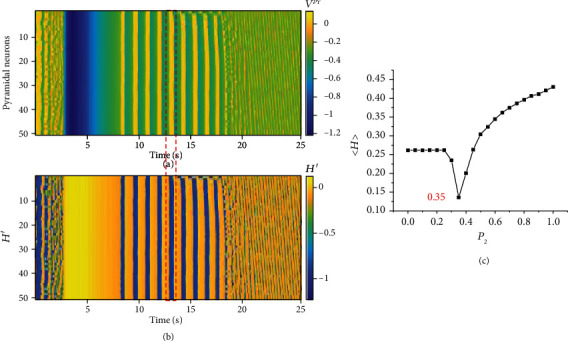
Variation in the energy consumption of the pyramidal neuronal population with the connection probability *P*_2_. (a) The firing pattern of the pyramidal neuronal population at *P*_2_ = 1. (b) The instantaneous power pattern of the energy consumption of the pyramidal neuronal population at *P*_2_ = 1. (c) The average energy consumption <H> of the pyramidal neuronal population changes with *P*_2_.

In this section, we studied the pyramidal neuronal population energy consumption variations with the change in the connection probability *P*_2_. The results are shown in Figure 5.

Figures 5(a) and 5(b) show the link between neuronal firing and energy consumption; the area in the red dotted frame shows that when seizure-like synchronous firing occurs in the neuronal population, the energy consumption also rises at the same time, which proves that epilepsy firing requires much energy. Figure 5(c) shows that the average energy consumption of the pyramidal neuronal population decreases first and then increases with increasing connection probability *P*_2_. This corresponds to the phenomenon shown in Figure 3(b), which further proves the close connection between neuronal firing and energy consumption, and verifies the regulation of astrocytes on neuronal population firing.

### 3.4. The Effect of *P* on the Synchronization of the Neuronal Network Connected by Electrical Synapses

**Figure 6:**
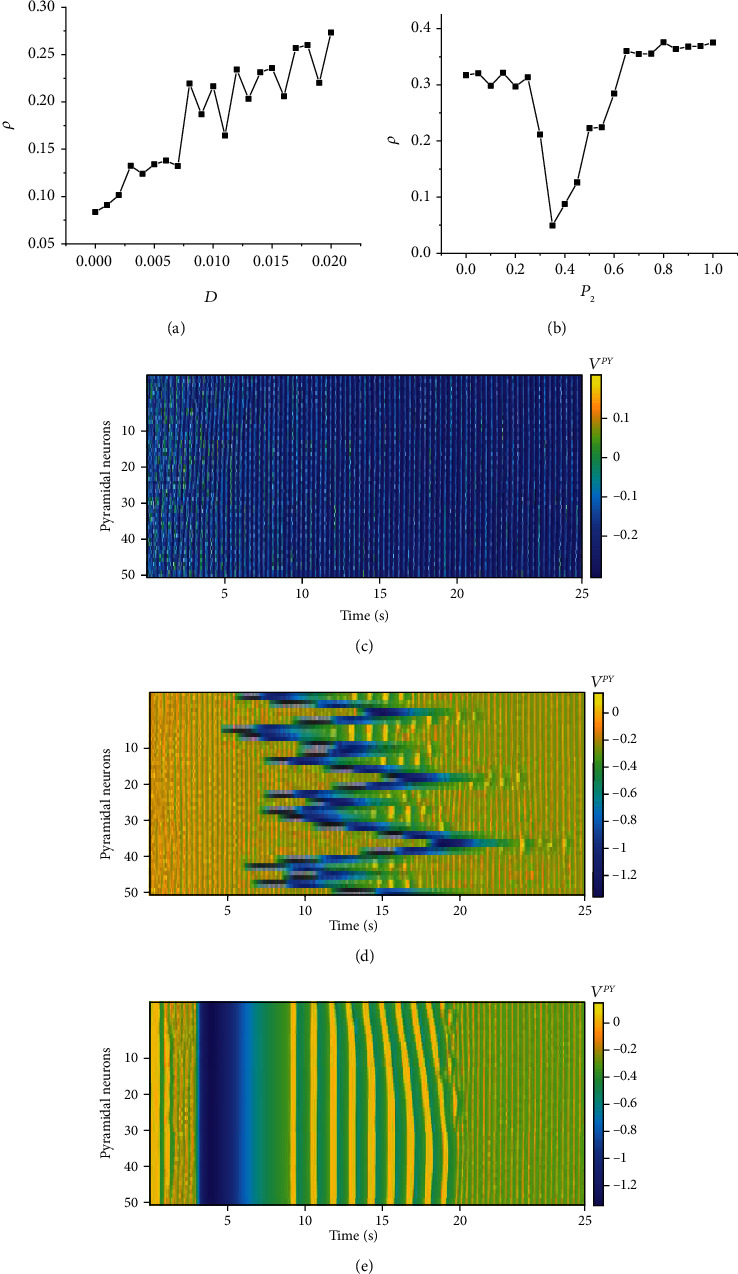
The impact of changes in *D* and *P*_2_ on the synchronous firing of the pyramidal neuronal population. (a) The change in the correlation coefficient *ρ* of the pyramidal neuronal population with the change in coupling strength *D*. (b) The change in the correlation coefficient *ρ* of the pyramidal neuronal population with the change in connection probability *P*_2_. (c) The time series of pyramidal neuronal population firing at *P*_2_ = 0. (d) The time series of pyramidal neuronal population firing at *P*_2_ = 0.35. (e) The time series of pyramidal neuronal population firing at *P*_2_ = 1.

In the nervous system of the brain, neurons are connected not only by chemical synapses but also by electrical synapses, and there are extensive electrical synaptic connections in the system [76]. Next, we studied the effect of the connection probability *P*_2_ on the firing activity of pyramidal neurons connected by electrical synapses, when chemical synapses are still used between interneurons and pyramidal neurons. The specific form of equation (12) is modified as follows:

(17) $$\begin{array}{c} I_{\text{syn},i}^{PY}\left(t\right)=D\left(v_{i-1}^{PY}\left(t\right)+v_{i+1}^{PY}\left(t\right)-2v_{i}^{PY}\left(t\right)\right)+g_{\text{si}}\left(g_{i-1}^{\text{IN}}\left(\text{t}\right)+g_{i}^{\text{IN}}\left(\text{t}\right)\right)\left(v_{i}^{PY}\left(t\right)-v_{\text{si}}\right), \end{array}$$

where *D* is the electrical synapse coupling strength. In this section, we studied the variations in the firing activity of the pyramidal neuronal population with the coupling intensity *D* when *g*_se_ = 0.1 and *P*_1_ = *P*_2_ = 0 and with the connection probability *P*_2_ when *g*_se_ = 0.1 and *D* = 0.02. The result is shown in Figure 6.

Figure 6(a) shows that the correlation coefficient *ρ* of the pyramidal neuronal population increases with the increase in the coupling strength *D*. The increase in *D* makes the postsynaptic neurons more sensitive to changes in the firing of presynaptic neurons, resulting in a change in the firing of the pyramidal neuronal population from asynchronous to synchronous; relative to that of chemical synapses, the synchronous firing shown in Figure 6(c) has no delay, which is consistent with the true characteristics of the electrical synapse and is more conducive to the firing synchronization of the neuronal network.

Figure 6(b) shows that the correlation coefficient *ρ* of the pyramidal neuronal population decreases first, corresponding to the process shown in Figure 6(c) and Figure 6(d), and then increases until *P*_2_ = 1; the firing of the neuronal population changes from asynchronous firing (Figure 6(d)) to seizure-like synchronous firing (Figure 6(e)).

The above results indicate that the coupling intensity *D* and the connection probability *P*_2_ play vital roles in the regulation of neuronal population synchronous firing activity, similar to those of the above chemical synapse study. The model is suitable for all synapse types (two types), and the results do not change with the connection method, proving the universality and stability of the feedback model.

## 4. Conclusion

An abnormal astrocyte structure may cause epilepsy, but few works have investigated the effects of astrocyte structural abnormalities such as gliosis on epilepsy through modeling methods. In this work, we used a new model to study the effects of astrocyte feedback on neuronal population firing and the generation and development of epilepsy in gliosis.

In the current research on the coupling model of neuron-astrocytes, most astrocytes are functional, and there is no comprehensive consideration of the actual spatial structure and relative distribution of astrocytes and neurons. In this work, we constructed a feedback model, which is very similar to the physiological structure, to describe the feedback coupling between astrocytes and neurons based on physiological and anatomical features.

We showed that the increase in the conductance *g*_se_ of the excitatory synapses strengthens the connection between pyramidal neurons, causing each neuron to be activated by the previous neuron and the firing state to become synchronized. More importantly, to study the transition of the firing state of the pyramidal neuronal population connected by chemical synapses, we changed the connection probability *P* and astrocyte feedback intensity *γ*_2_, and the result showed that the participation of astrocytes in neuronal firing activities will lead to the transition of the neuron firing state from a synchronous state with slight depolarization block firing to asynchronous firing with slight local seizure-like firing, when astrocytes are in gliosis and gradually become more severe with an increase in *P*_2_, causing astrocyte feedback to become abnormal and leading to the transition of neurons from asynchronous firing to seizure-like synchronous firing. Additionally, the analysis of the firing activity of the neuron population showed that the transition of neuronal firing activity is regulated by Ca^2+^. Then, we analyzed the energy consumption of pyramidal neurons connected by chemical synapses and the firing state of pyramidal neurons connected by electrical synapses according to the connection probability *P*_2_, and the results further confirmed the influence of astrocyte feedback on neuronal firing activity and the universality and stability of the feedback model. Therefore, the results of this study demonstrate the ability of the astrocyte population to regulate the firing of neurons and the key role of astrocyte network gliosis in neuronal seizures. In summary, our results reveal a potential mechanism of seizure firing and provide a new direction for the treatment of brain diseases such as epilepsy.

## Figures and Tables

**Table 1:** Parameters used in the model.

Parameter	Value	Parameter	Value	Parameter	Value
C_m_	1.0	*v* ^∗^	-0.22	*V*_3_	0.9 *μ*Ms^−1^
*g*_*ca*_	1.0	*α*	0.001	*k*_3_	0.1 *μ*M
*v*_*ca*_	1.0	*σ*_s_	0.02	*d*_1_	0.13 *μ*M
*g*_*k*_	2.0	*α*_*s*_	0.1	*d*_5_	0.082 *μ*M
*v*_*k*_	-0.7	*β*_*s*_	0.05	*a*_2_	0.2 *μ*Ms^−1^
*g*_*l*_	0.5	*g*_si_	0.1	*d*_2_	1.05 *μ*M
*v*_*l*_	-0.5	*v*_se_	-0.85	*d*_3_	0.94 *μ*M
*v*_1_	-0.01	*v*_si_	0	*c*_0_	2 *μ*M
*v*_2_	0.15	IP_3_^∗^	0.16 *μ*M	*τ*_Ca^2+^_	6 s
*v*_3_	0.1	*τ*_*ip*_3__	7 s	[Ca]_th_	0.2
*v*_4_	0.145	*r*_*ip*_3__	7.2 *μ*Ms^−1^	*κ*	0.5 s^−1^
∅	1.15	*c*_1_	0.185	*k*_*g*_	0.1
*θ*_*s*_	0.2	*V*_1_	6 s^−1^	*γ*_1_	0.05
*ε*	0.0005	*V*_2_	0.11 s^−1^	*P*_1_	0.8

## Data Availability

The data used to support the findings of this study are available from the corresponding author upon request.
